# Enteral nutrition practices and associated outcomes in preterm infants with a gestational age less than 34 weeks: a single-center retrospective study

**DOI:** 10.3389/fnut.2026.1760487

**Published:** 2026-06-04

**Authors:** Jian-Hong Liu, Zhen Hu, Jin-Xue Pan, Hao He, Xiao-Qian Yi, Xiao-Fang Zhu

**Affiliations:** Department of Neonatology, Jingzhou Hospital Affiliated to Yangtze University, Jingzhou, China

**Keywords:** bronchopulmonary dysplasia, enteral nutrition, feeding intolerance, preterm infant, rate of feeding increase, total enteral nutrition time

## Abstract

**Objective:**

This study aims to assess the current status of enteral nutrition (EN) in preterm infants with a gestational age (GA) less than 34 weeks at a single neonatal intensive care unit (NICU) and to identify factors associated with delayed achievement of total enteral nutrition (TEN).

**Methods:**

A retrospective analysis was conducted on 137 preterm infants with GA less than 34 weeks admitted to the NICU of Jingzhou Hospital Affiliated to Yangtze University between December 2019 and June 2024. Infants were categorized into Group A (GA < 32 weeks) and Group B (GA ≥ 32 weeks). Each group was further divided into subgroups based on whether TEN was achieved within 21 days (Group A1) or not (Group A2), and within 14 days (Group B1) or not (Group B2), respectively. Differences in EN practices and clinical complications were compared using *t*-tests, rank sum tests, chi-squared tests, or Fisher’s exact tests.

**Results:**

The initiation of enteral feeding within 48 h and 24 h after birth occurred in 97.08% and 46.72% of cases, respectively. The mean time to achieve TEN was 42.50±22.06 days for infants with GA 26^+0^–27^+6^ weeks, 28.69±9.78 days for GA 28^+0^–31^+6^ weeks, and 17.27±0.70 days for GA 32^+0^–33^+6^ weeks. The median time to achieve TEN was 21 days in Group A and 14 days in Group B. Initial feeding volumes in Groups A1 (11.35 mL/kg·d) and B1 (15.05 mL/kg·d) were significantly higher than those in Groups A2 (6.09 mL/kg·d) and B2 (12.77 mL/kg·d), respectively. A more rapid advancement of feeding volume during the first one to 2 weeks after birth was also observed in Groups A1 and B1 (*p* < 0.05 or 0.01). The incidence of bronchopulmonary dysplasia (BPD) in Group A1 and feeding intolerance in Group B1 were lower compared to their respective control groups (*p* < 0.05).

**Conclusion:**

Preterm infants with GA less than 34 weeks showed a delay in the achievement of TEN. Higher initial feeding volumes and a faster rate of feeding advancement within the first 2 weeks after birth were associated with earlier achievement of TEN and a lower incidence of BPD.

## 1 Introduction

With the increasing survival rate of preterm infants in China, the role of nutrition in facilitating postnatal catch-up growth has gained increasing clinical relevance. Nutrition is the core of survival, growth, and development for preterm infants after birth, and a decisive factor in achieving catch-up growth, promoting the maturation of organ development, and reducing the risk of complications (1). The incidence of malnutrition and related complications remains high due to the immaturity of multiple organ systems in preterm infants and the compounded effects of associated morbidities. The risk of malnutrition-related diseases in early preterm infants is significantly higher than that in late preterm infants and full-term infants (1). In the short term, it elevates the risk of infectious diseases, extrauterine growth restriction (EUGR), bronchopulmonary dysplasia (BPD), neonatal necrotizing enterocolitis (NEC), retinopathy of prematurity (ROP), and other morbidities [1, SDC 1, 6] (2), prolongs hospital stay, and increases the consumption of medical resources. In the long term, there is an increased risk of neurological sequelae and long-term metabolic diseases [1, SDC 1] (3). Nutritional support for preterm infants with a gestational age of < 34 weeks faces core challenges related to feeding advancement rate and safety (1). A reasonable initiation and progression protocol for enteral nutrition is a key strategy to reduce complications in preterm infants (3). Although individualized enteral nutrition (EN) protocols have been implemented, challenges persist, particularly among very low birth weight infants (VLBWI) and extremely low birth weight infants (ELBWI), who continue to exhibit elevated rates of extrauterine growth restriction (EUGR), feeding intolerance (FI), and other complications within the neonatal intensive care unit (NICU) setting (4). Nevertheless, there is a lack of gestational age (GA)–stratified data on the time required to achieve full enteral nutrition (TEN). Using real-world Chinese clinical data, this study investigated risk factors for delayed transition to TEN, evaluated the applicability of international guidelines, and examined the relationship between nutrition and BPD in preterm infants born at less than 34 weeks’ gestation, in order to inform individualized feeding strategies.

## 2 Materials and methods

### 2.1 Study population

A retrospective study was conducted on preterm infants with a GA less than 34 weeks who were admitted to the NICU of Jingzhou Hospital Affiliated to Yangtze University between December 2019 and June 2024.

Inclusion criteria: (1) Delivery in the obstetrics department of Jingzhou Hospital Affiliated to Yangtze University, with a GA less than 34 weeks. (2) Admission to the NICU within 24 h after birth. Exclusion criteria: (1) Presence of specific conditions, including inherited metabolic diseases, cyanotic congenital heart disease, congenital gastrointestinal malformations, or conditions requiring surgical intervention. (2) Withholding or withdrawing treatment, and voluntary discharge or death. (3) Incomplete medical records. (4) Infants who were transferred from other hospitals or admitted more than 24 h after birth.

The study was approved by the Ethics Committee of Jingzhou Hospital Affiliated to Yangtze University (Approval No. 2024-139-01). Written informed consent for participation in this study was provided by the participants’ legal guardians/next of kin.

### 2.2 Study methods

#### 2.2.1 Grouping

All enrolled infants were classified into two groups according to GA: Group A (GA < 32 weeks) and Group B (GA ≥ 32 weeks). Group A was further divided based on the time required to achieve TEN into Group A1 (TEN achieved within 21 days) and Group A2 (TEN achieved after 21 days). Group B was similarly subdivided into Group B1 (TEN achieved within 14 days) and Group B2 (TEN achieved after 14 days).

#### 2.2.2 Data collection

Medical records of the enrolled infants were retrospectively retrieved from the hospital’s information system database. The following data were collected: (1) Maternal characteristics: diabetes mellitus, hypertension, thyroid disorders (hyperthyroidism/hypothyroidism), liver disease, and prenatal intraamniotic infection. (2) Neonatal characteristics: sex, GA, birth weight (BW), mode of delivery, and Apgar scores. (3) EN parameters: time to initiation of feeding, initial feeding volume, rate of feeding increase, type of dairy product, feeding method, feeding volume at discharge, and length of hospital stay. (4) Complications: gastroesophageal reflux (GER), FI, necrotizing enterocolitis (NEC), sepsis, retinopathy of prematurity (ROP), bronchopulmonary dysplasia (BPD), neonatal hypothyroidism, cholestasis, gastrointestinal bleeding, gastrointestinal perforation, neonatal respiratory distress syndrome (RDS), mechanical ventilation (including invasive and/or non-invasive ventilation during hospitalization), and pulmonary surfactant (PS), or hemodynamically significant patent ductus arteriosus (hsPDA). (5) Parenteral nutrition (PN) and discharge outcomes: time to initiation of PN, duration of PN, and body weight at discharge.

#### 2.2.3 Diagnostic criteria

1. Main outcome measures

TEN: Considered achieved when either an EN volume of 150 mL/(kg·d) (3) or enteral caloric intake of 120 kcal/(kg·d) (5) was reached. Calculation method of caloric intake: Daily caloric intake = (Total milk volume in 24 h × Calories per 100 mL of milk) / kg. The dairy products and their caloric values used during hospitalization were as follows: human milk (67 kcal/100 mL), human milk fortifier (73 kcal/100 mL for half-strength fortification, 80 kcal/100 mL for full-strength fortification), preterm infant formula (80 kcal/100 mL), term infant formula (67 kcal/100 mL), and special formula milk powders: Peptamen Junior (67 kcal/100 mL, can be reconstituted to different caloric concentrations), Neocate (67 kcal/100 mL), and Nutrison Energy (100 kcal/100 mL) (1 kcal = 4.184 kJ).

Feeding initiation was prioritized with human milk; formula was used if human milk was unavailable. The regimen was selected and adjusted by clinicians based on gestational age, body weight, feeding status, and tolerance.

1. Secondary outcome measures: incidence of enteral nutrition-related complications in preterm infants.

FI diagnostic criteria (6, 7): Diagnosis was based on one or more of the following: (1) Vomiting ≥ 3 times/day; (2) No increase or a decrease in feeding volume for > 3 days; (3) Vomiting or gastric residuals with a coffee-ground appearance, with or without positive fecal occult blood; (4) Residual milk exceeding 50% of the previous feeding volume; (5) Abdominal distension with an increase in abdominal circumference ≥ 1.5 cm within 24 h, accompanied by an abnormal intestinal pattern.

BPD: Diagnosed according to the 2018 criteria published by the Eunice Kennedy Shriver National Institute of Child Health and Human Development (8).

Cholestasis: Defined as direct bilirubin ≥ 34.2 μmol/L (or 2 mg/dL) following PN administration for more than 14 days, excluding cholestasis caused by other factors such as biliary atresia or hereditary metabolic disorders (9).

Other neonatal complications: Diagnostic criteria for NEC [6]633–636, sepsis [6]513, ROP [6]1,027–1,028, GER [6]620–621, neonatal hypothyroidism [6]923, gastrointestinal bleeding [6]427–428, gastrointestinal perforation [6]651, Neonatal Respiratory Distress Syndrome [6]575–577 and hsPDA [6]743–744 were referenced from the 5th edition of Practice of Neonatology.

1. Other indicators: time to regain birth weight, length of hospital stay, duration of parenteral nutrition use, etc.

Weight gain velocity during hospitalization: Calculated using the formula: Weight gain velocity (g/(kg·d)) = [1,000 × ln(discharge weight/BW) / (age at discharge − days of life when BW was regained)] (10).

1. Maternal diseases during pregnancy: diagnostic criteria were referenced from the 8th edition of obstetrics and gynecology (11).
2. Special Note: Enrolled infants with BPD received fluid restriction but not calorie restriction. Clinicians adhered to both domestic and international feeding guidelines, individually determining the timing of feeding initiation and feeding strategies based on each infant’s respiratory status, hemodynamic stability, and gastrointestinal tolerance. In critically ill infants with unstable respiratory and circulatory conditions, feeding initiation was appropriately delayed. Furthermore, the severity of illness was positively correlated with the duration of the feeding delay. Based on national and international guidelines, BPD infants received individualized nutrition support, gradually transitioning from tube feeding (< 32 weeks GA) to oral feeding via gravity, intermittent, or continuous pump infusion, balancing respiratory and nutritional needs to ensure safety and adequate intake. All infants diagnosed with RDS in this study received respiratory support, including invasive or non-invasive mechanical ventilation. Pulmonary surfactant (PS, 200 mg/kg) was administered as needed. The first dose was uniformly given at 200 mg/kg. The indications and timing of surfactant treatment were consistent with the 2022 European Guidelines for RDS (12). This study only recorded whether PS was administered (yes/no); data on the frequency of repeat doses or cumulative dosage were not collected.
3. Grouping: Given the lack of a standardized time to achieve TEN in preterm infants, and referring to domestic and international guidelines, infants were grouped by time to TEN: 14 days (Group B) and 21 days (Group A) (7, 13–15).

### 2.3 Statistical analysis

Statistical analyses were conducted using SPSS version 22.0. Continuous variables with a normal distribution were presented as mean ± standard deviation (*x–* ± *s*), while those not following a normal distribution were expressed as the median and interquartile range, M (Q1, Q3). Between-group comparisons for continuous variables were performed using the independent samples *t*-test or the rank sum test (Mann–Whitney *U* test), as appropriate. Categorical variables were summarized as frequencies and percentages, and between-group comparisons were carried out using the chi-squared ( $\chi^{2}$ ) test or Fisher’s exact test. A *p*-value < 0.05 was considered statistically significant.

## 3 Results

### 3.1 Final enrollment and grouping

**Figure 1:**
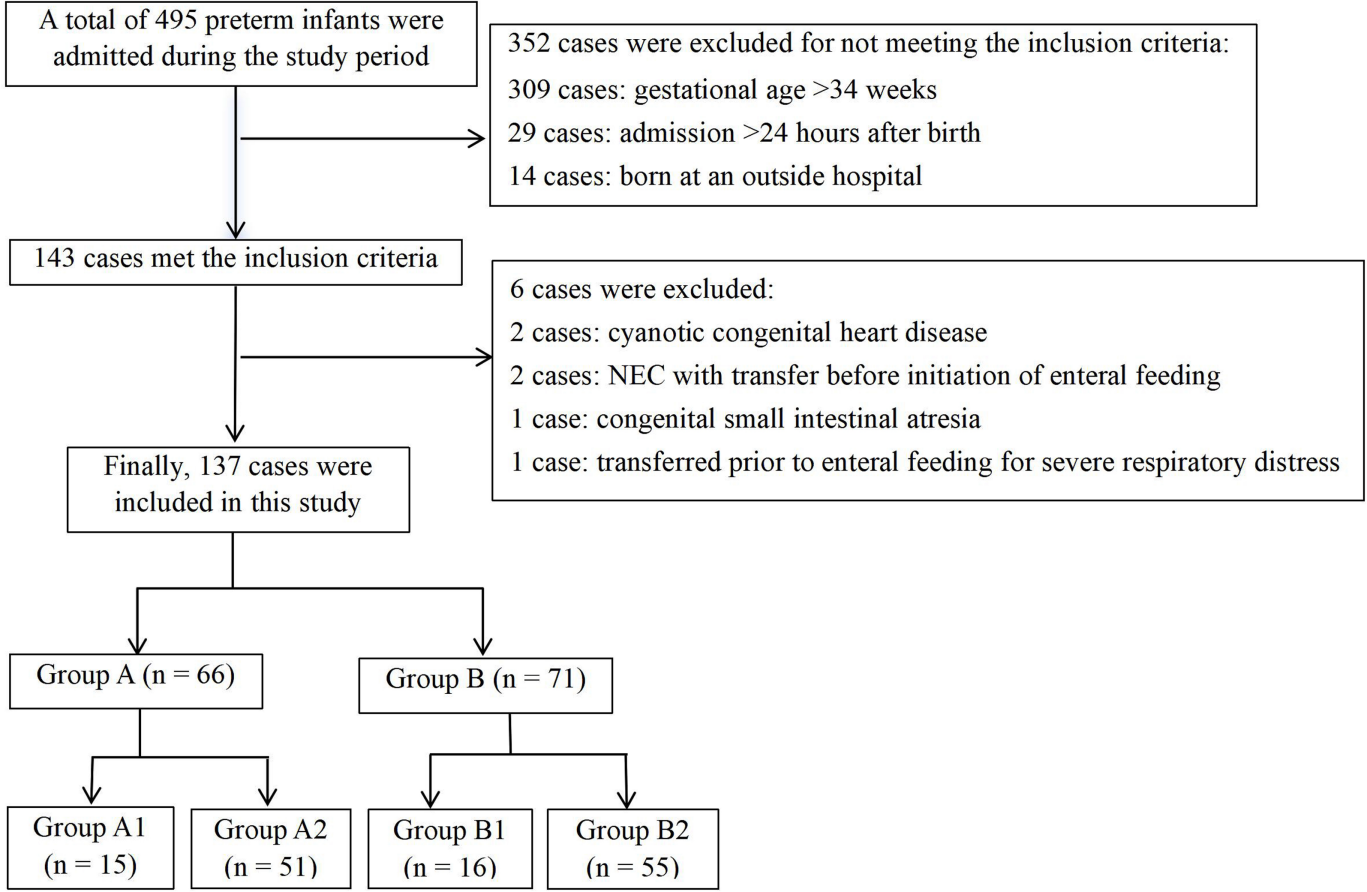
Flowchart of inclusion and grouping of preterm infants in the present study.

During the study period, 495 preterm infants (Infants with GA < 37 weeks) were admitted to the NICU. A total of 161 preterm infants met the inclusion criteria, and 24 were excluded; ultimately, 137 infants were enrolled in the study. Classification by GA was carried out, with Group A comprising infants with GA < 32 weeks and Group B comprising those with GA ≥ 32 weeks. Group A was further subdivided into Group A1 and Group A2 based on whether TEN was achieved within 21 days. Similarly, Group B was subdivided into Group B1 and Group B2 according to whether TEN was achieved within 14 days (see Figure 1).

Group A had a longer hospital stay and required more time to achieve full enteral feeding compared to Group B. Subgroup analyses were conducted separately within each group during hospitalization.

### 3.2 General characteristics

No statistically significant differences were observed between Groups A1 and A2 or between Groups B1 and B2 with respect to sex, GA, BW, mode of delivery (cesarean section rate), 1-min Apgar score ≤ 7, or 5-min Apgar score ≤ 7 (*p* > 0.05) (see Table 1).

**Table 1:** Comparison of general characteristics and maternal diseases during pregnancy among study groups.

Item		Group A1^†^	Group A2^†^	*χ*^2^/*t*	*p*	Group B1^†^	Group B2^†^	*χ* ^2^	*p*
Sex	Male [*n* (%)]	11 (73.3)	28 (54.9)	1.629	0.202	6 (37.5)	35 (63.6)	3.470	0.062
	Female [*n* (%)]	4 (26.7)	23 (45.1)			10 (62.5)	23 (36.4)		
GA (weeks)		30^+4^ (30^+0^, 31^+5^)^‡^	30^+2^ (29^+1^, 31^+0^)^‡^	−1.387	0.168	33^+1^ (32^+5^, 33^+3^)^‡^	33^+0^ (32^+3^, 33^+4^)^‡^	−0.635	0.531
BW (g)		1,425 (1,280, 1,640)^‡^	1,320 (1,170, 1,540)^‡^	1.518	0.134	1,765 (1,609, 1936)^‡^	1,770 (1,620, 2,100)^‡^	−0.453	0.652
Cesarean section [*n* (%)]		7 (46.7)	22 (43.1)	0.059	0.809	15 (93.8)	41 (74.5)	1.712	0.191
1-min Apgar score ≤ 7 [*n* (%)]		2 (13.3)	11 (21.6)	0.113	0.737	1 (6.3)	5 (9.1)	-	1.000^a^
5-min Apgar score ≤ 7 [*n* (%)]		1 (6.7)	1 (2.0)	0.006	0.406^a^	2 (12.5)	0 (0.0)	3.245	0.072
Diabetes mellitus [*n* (%)]		2 (13.3)	4 (7.8)	0.019	0.889	2 (12.5)	17 (31.5)	1.391	0.238
Hypertension [*n* (%)]		4 (26.7)	6 (11.8)	1.011	0.315	5 (31.3)	11 (20.0)	0.370	0.543
Thyroid disease [*n* (%)]		1 (6.7)	5 (9.8)	–	1.000^a^	0 (0.0)	4 (7.3)	–	0.568^a^
Intra-amniotic infection [*n* (%)]		0 (0.0)	3 (5.9)	–	1.000^a^	1 (6.3)	6 (10.9)	0.005	0.941
Liver disease [*n* (%)]		2 (13.3)	4 (7.8)	0.019	0.889	0 (0.0)	3 (5.5)	–	1.000^a^

^a^Fisher’s exact test; *p* < 0.05 indicates statistical significance. ^†^*n* = 15, 51, 16, 55 (sample size of each group); ^‡^Data are presented as median (Q1, Q3).

### 3.3 Maternal diseases during pregnancy

No statistically significant differences were identified between Groups A1 and A2 or between Groups B1 and B2 in terms of maternal comorbidities during pregnancy, including diabetes mellitus, hypertension, thyroid disease, liver disease, or intra-amniotic infection (*p* > 0.05) (see Table 1).

### 3.4 EN status

In this study, the utilization rate of human milk was 80.3% (110/137), among which mixed feeding accounted for 62.8% (86/137), and exclusive human milk feeding only accounted for 17.5% (24/137). No statistically significant difference was observed in the time to initiation of feeding between Groups A1 and A2 (*p* > 0.05). However, Group A1 demonstrated a higher initial feeding volume and a more rapid rate of feeding advancement during weeks 1 and 2 compared to Group A2. The length of hospital stay was significantly longer in Group A2, with differences reaching statistical significance (*p* < 0.05). A gradual decline in the feeding advancement rate was noted in Group A1 during weeks 3 and 4 compared to the initial 2 weeks. Conversely, the rate of feeding advancement in Group A2 during week 4 was higher than that in Group A1 (*p* < 0.01), although a decreasing trend was observed in both groups overall. Statistically significant differences were identified between the groups in the time to achieve TEN and length of hospital stay (*p* < 0.05), while no significant differences were found in the remaining parameters (*p* > 0.05).

Similarly, no statistically significant difference was observed in the time to initiation of feeding between Groups B1 and B2 (*p* > 0.05). Group B1 exhibited a higher initial feeding volume, and a faster rate of feeding advancement during weeks 1 and 2 in comparison to Group B2, with statistically significant differences (*p* < 0.05). However, the feeding advancement rate in Group B1 during week 3 was lower than that in Group B2 (*p* < 0.05). Significant differences were observed between the groups in time to achieve TEN and discharge weight (*p* < 0.05), while no significant differences were observed for the other parameters (*p* > 0.05) (see Table 2).

**Table 2:** Comparison of EN status among study groups.

Item	Group A1^†^	Group A2^†^	*Z*/*T*	*p*
Time to initiation of feeding (h)	24.6 (20.41, 27.45)^‡^	23.07 (17.85, 30.00)^‡^	−0.245	0.811
Initial feeding volume [mL/(kg·d)]	11.35 (8.89, 12.09)^‡^	6.09 (4.90, 8.57)^‡^	−3.366	0.000^*^
Week 1 daily average feeding advancement rate [mL/(kg·d)]	9.74 (6.32, 11.82)^‡^	3.63 (1.86, 6.86)^‡^	−3.864	0.000^*^
Week 2 daily average feeding advancement rate [mL/(kg·d)]	9.83 (8.10, 12.29)^‡^	7.35 (5.65, 9.23)^‡^	3.340	0.001^*^
Week 3 daily average feeding advancement rate [mL/(kg·d)]	4.52 (1.95, 7.42)^‡^	6.18 (3.76, 8.30)^‡^	−1.229	0.225
Week 4 daily average feeding advancement rate [mL/(kg·d)]	−1.20 (−2.12, 0.00)^‡^	4.74 (2.40, 7.27)^‡^	−3.994	0.000^*^
Week 5 daily average feeding advancement rate [mL/(kg·d)]	0.78 (−1.38, 2.06)^‡^	4.03 (1.46, 6.76)^‡^	−1.751	0.088
TEN time (d)	17.00 (16.00, 20.00)^‡^	33.00 (27.00, 36.50)^‡^	−5.716	0.000^*^
Average feeding velocity during hospitalization [mL/(kg·d)]	4.07 (2.90, 7.78)^‡^	5.02 (3.42, 6.36)^‡^	0.068	0.946
Average growth velocity during hospitalization [g/(kg·d)]	14.91 (12.62, 17.07)^‡^	15.39 (13.99, 17.06)^‡^	−0.276	0.783
Feeding volume at discharge [mL/(kg·d)]	143.59 (139.81, 147.54)^‡^	148.45 (137.34, 160.00)^‡^	0.076	0.940
Discharge weight (g)	2,185 (1,995, 2,340)^‡^	2,350 (2,090, 2,530)^‡^	−1.505	0.137
Length of hospital stay (d)	33 (22, 41)^‡^	41 (32, 57)^‡^	−2.197	0.014^*^

Item	Group B1^†^	Group B2^†^	*Z*/*T*	*p*
Time to initiation of feeding (h)	25.85 (23.59, 26.87)^‡^	24.72 (16.5, 26.37)^‡^	−1.837	0.066
Initial feeding volume [mL/(kg·d)]	15.05 (11.52, 16.16)^‡^	12.77 (9.52, 14.68)^‡^	−2.347	0.018^*^
Week 1 daily average feeding advancement rate [mL/(kg·d)]	13.21 (11.40, 15.09)^‡^	11.17 (6.22, 13.88)^‡^	−2.147	0.031^*^
Week 2 daily average feeding advancement rate [mL/(kg·d)]	12.06 (11.16, 13.88)^‡^	9.86 (8.37, 11.01)^‡^	4.213	0.000^*^
Week 3 daily average feeding advancement rate [mL/(kg·d)]	0.65 (−1.42, 0.93)^‡^	5.05 (2.19, 8.07)^‡^	−2.799	0.009^*^
TEN time (d)	13.50 (12.25, 14.00)^‡^	18.00 (15.00, 23.75)^‡^	−5.716	0.000^*^
Average feeding velocity during hospitalization [mL/(kg·d)]	11.72 (7.63, 13.72)^‡^	9.52 (6.50, 12.26)^‡^	1.226	0.224
Average growth velocity during hospitalization [g/(kg·d)]	14.24 (12.16, 16.04)^‡^	15.85 (13.94, 17.72)^‡^	−0.276	0.783
Feeding volume at discharge [mL/(kg·d)]	157.26 (147.60, 164.77)^‡^	150.00 (133.99, 156.52)^‡^	0.076	0.940
Discharge weight (g)	2,080 (2,001, 2,221)^‡^	2,215 (2,130, 2,360)^‡^	−2.762	0.007^*^
Length of hospital stay (d)	17 (16, 24)^‡^	20 (12, 28)^‡^	−0.803	0.427

^*^p < 0.05 indicates statistical significance. ^†^*n* = 15, 51, 16, 55 (sample size of each group); ^‡^Data are presented as median (Q1, Q3).

### 3.5 PN status

The duration of PN was significantly longer in Group A2 compared to Group A1 (*p* < 0.05), and in Group B2 compared to Group B1 (*p* < 0.05). No statistically significant differences were observed between the groups in the time to initiation of PN (*p* > 0.05) (see Table 3).

**Table 3:** Comparison of PN status among study groups.

Item	Group A1^†^	Group A2^†^	*Z*/*T*	*p*
Time to initiation of PN (h)	18.75 (2.52, 23.17)^‡^	16.85 (6.77, 22.85)^‡^	−0.137	0.891
Duration of PN (d)	14.00 (11.00, 19.00)^‡^	26.00 (20.00, 36.00)^‡^	−4.395	0.000^*^

Item	Group B1^†^	Group B2^†^	*Z*/*T*	*p*
Time to initiation of PN (h)	24.20 (4.17, 26.55)^‡^	18.45 (8.17, 25.20)^‡^	−1.209	0.231
Duration of PN (d)	10.00 (9.00, 10.75)^‡^	12.00 (9.00, 16.00)^‡^	−3.538	0.001^*^

PN, parenteral nutrition; ^*^p < 0.05 indicates statistical significance. ^†^*n* = 15, 51, 16, 55 (sample size of each group); ^‡^Data are presented as median (Q1, Q3).

### 3.6 Complications

A higher incidence of BPD was observed in Group A2 compared to Group A1 (*p* < 0.05), Group A2 had a higher PS utilization rate than Group A1 (p < 0.05), while no other parameters showed statistically significant differences (*p* > 0.05). In Group B2, the incidence of FI was higher than in Group B1 (*p* < 0.05), with no statistically significant differences in the remaining parameters (*p* > 0.05) (see Table 4).

**Table 4:** Comparison of complication incidence among study groups [*n* (%)].

Item	Group A1^†^	Group A2^†^	*χ* ^2^	*p*
FI	10 (66.7)	40 (78.4)	0.350	0.554
NEC	1 (6.7)	3 (5.9)	–	1.000^a^
Sepsis	7 (46.7)	27 (52.9)	0.183	0.669
ROP	3 (20.0)	22 (43.1)	2.637	0.104
BPD	0 (0)	18 (35.3)	–	0.007^*,a^
Hypothyroidism	2 (13.3)	9 (17.6)	0.000	1.000
Cholestasis	2 (13.3)	6 (11.8)	0.000	1.000
Gastrointestinal perforation or bleeding	1 (6.7)	11 (21.6)	0.874	0.350
hsPDA	2 (13.3)	10 (19.6)	–	0.719^a^
Respiratory distress syndrome	12 (80.0)	48 (94.1)	–	0.125
Mechanical ventilation	12 (80.0)	48 (94.1)	–	0.125
Pulmonary surfactant	5 (33.3)	33 (64.7)	–	0.040^*^

Item	Group B1^†^	Group B2^†^	*χ* ^2^	*p*
FI	1 (6.3)	21 (38.2)	4.511	0.034^*^
Sepsis	3 (18.8)	6 (10.9)	0.162	0.687
ROP	0 (0)	1 (1.8)	–	1.000^a^
Hypothyroidism	2 (11.5)	5 (9.1)	0.000	1.000
Cholestasis	0 (0)	4 (7.3)	–	0.568^a^
Gastrointestinal perforation or bleeding	0 (0)	4 (7.3)	–	0.568^a^
GER	0 (0)	2 (3.6)	0.000	1.000
hsPDA	1 (6.3)	4 (7.3)	–	1.000
Respiratory distress syndrome	9 (56.3)	28 (50.9)	–	0.781
Mechanical ventilation	9 (56.3)	28 (50.9)	–	0.781
Pulmonary surfactant	5 (31.3)	16 (29.1)	–	1.000

FI, feeding intolerance; NEC, neonatal necrotizing enterocolitis; BPD, bronchopulmonary dysplasia; GER, gastroesophageal reflux; ROP, retinopathy of prematurity; ^*^p < 0.05 indicates statistical significance; ^a^Fisher’s exact test. ^†^*n* = 15, 51, 16, 55 (sample size of each group); ^‡^Data are presented as median (Q1, Q3). Mechanical ventilation includes invasive mechanical ventilation and non-invasive ventilation.

## 4 Discussion

Lower GA in preterm infants is associated with greater physiological immaturity, increased risk of complications, and heightened difficulty in establishing EN. Several studies have demonstrated a strong association between suboptimal EN practices in preterm infants and adverse outcomes, including EUGR, BPD, metabolic abnormalities, and delayed neurodevelopment (1–3, 16, 17). Although multiple national and international guidelines and recommendations for EN in preterm infants have been developed, clinical observations have indicated that the time required to achieve TEN in extremely and moderately preterm infants in China remains longer than that reported in developed countries with a comparatively higher incidence of EN-related complications.

### 4.1 Delayed initiation of feeding and low initial feeding volume in preterm infants

Early initiation of enteral feeding promotes the release of endogenous nutrients, supports gastrointestinal mucosal development and maturation, and stimulates digestive enzyme activity, thereby enhancing nutrient absorption. Additionally, early feeding reduces the risk associated with prolonged fasting and contributes to the earlier achievement of TEN (18). In this study involving 137 preterm infants, the median time to initiation of feeding was 24.7 h, with a range from 2.4 to 67.4 h. This median time exceeded the 24-h target recommended by the 2024 Expert Consensus on Enteral Nutrition Management in Preterm Infants (3). Among the 137 infants, 46.72% (64/137) initiated feeding within 24 h after birth, and 97.08% (133/137) within 48 h. These rates were lower than the 70% reported in a 2021 Chinese network survey (19).

Current guidelines suggest that delayed initiation may be appropriate in the presence of complications such as asphyxia, respiratory distress, sepsis, hypotension, mechanical ventilation, umbilical catheterization, or the need for invasive procedures (7). In this study, all infants who did not receive feeding within 24 h presented with at least one of these conditions. Among them, those with more severe complications such as acute respiratory distress syndrome, hemodynamic instability, or early gastrointestinal bleeding had feeding initiation delayed to beyond 48 h. Relevant research on this topic remains limited in China. Li et al. (13) reported a feeding initiation time of (20.91 ± 11.88) h in tolerant and (29.20 ± 34.66) h in intolerant preterm infants < 32 weeks’ GA. These results align with our findings and represent the current situation in China. The extent to which these timings relate specifically to critical illness and high FI incidence must be clearly established, emphasizing the importance of further large-sample research to identify the underlying factors.

Initiating feeding as early as possible after birth can not only activate intestinal function at the earliest opportunity but also rapidly trigger the secretion of gastrointestinal hormones such as gastrin and cholecystokinin in preterm infants through the mechanical stimulation of sucking movements, thereby enhancing the activity of digestive enzymes (20). A randomized controlled trial (RCT) conducted by Razzaghy et al. reported that initiating feeding within 36 h of birth increased the number of days with TEN during the neonatal period in infants with GA < 32 weeks (21). Similarly, findings from Gao et al. demonstrated that feeding initiation within 24 h after birth shortened the time to achieve TEN and improved outcomes in preterm infants with GA < 32 weeks (22).

Both the practice of early feeding initiation and the initial feeding volume are key factors affecting TEN attainment, with distinct functional dimensions and a synergistic effect. Although subgroup analysis by Chitale et al. (23) suggested that the timing of EN initiation is more important than the initial feeding volume, initial feeding volume establishes the “starting efficiency” of nutritional support. In this study, initial feeding volumes in groups A1 and B1 (who achieved TEN on schedule) were markedly greater than those in groups A2 and B2 (who did not), within guideline-recommended ranges. This indicates that an optimal initial feeding volume may accelerate the time to reach the target nutrient intake.

The present findings indicate that delayed achievement of TEN in preterm infants with GA < 34 weeks may be associated with lower initial feeding volumes. In Group A, the average initial feeding volume was 7.13 (5.15, 11.30) mL/(kg·d), which was lower than volumes reported in the Guangdong region (13). Within Group A1, the initial feeding volume was 11.35 (8.89, 12.09) mL/(kg·d), meeting the 2015 guideline range of 10–15 mL/(kg·d), though slightly below the 12–24 mL/(kg·d) range recommended in both the 2022 European guidelines and the 2024 Chinese guidelines (1, 3, 7). The initial feeding volume in Group A2 was 6.09 (4.90, 8.57) mL/(kg·d), which fell significantly below guideline recommendations. The difference in initial feeding volume between Groups A1 and A2 was statistically significant (*p* < 0.05). In Group B, the average initial feeding volume was 13.41 (9.82, 15.05) mL/(kg·d), aligning approximately with guideline recommendations. Group B1 had an initial feeding volume of 15.05 (11.52, 16.16) mL/(kg·d), while Group B2 had 12.77 (9.52, 14.68) mL/(kg·d), with a statistically significant difference between the two subgroups (*p* < 0.05).

An RCT by Razzaghy et al. involving 102 preterm infants with GA between 28^0/7^–32^6/7^ weeks indicated that those receiving 60–80 mL/(kg·d) within 36 h after birth achieved TEN earlier than those receiving 20–30 mL/(kg·d), which is consistent with the findings of this study (21). However, research on the relationship between initial feeding volume and time to achieve TEN in preterm infants with GA between 32 and 34 weeks remains limited. Further large-scale studies are warranted to confirm this relationship.

### 4.2 Faster rate of feeding advancement during the first 2 weeks after birth reduces time to achieve TEN in preterm infants

The findings of this study indicated that the average rate of feeding advancement during the first 2 weeks after birth was higher in Groups A1 and B1 compared to Groups A2 and B2, respectively. This suggests that the rate of feeding advancement during the initial postnatal period plays a key role in achieving TEN in preterm infants with a GA < 34 weeks. Overall, the rate of feeding increase observed in preterm infants in this study was lower than the 2015 guideline recommendation of 15–20 mL/(kg·d) for infants with BW < 1,000 g, and also lower than the recommendation of 18–30 mL/(kg·d) by the European Society for Paediatric Gastroenterology, Hepatology and Nutrition (1, 7). This discrepancy may be attributable to the relatively high incidence of FI, leading clinicians to adopt more conservative feeding protocols.

Earlier achievement of TEN in Groups A1 and B1, which received a more rapid feeding advancement, is consistent with findings reported by Montealegre et al. and Dorling et al. (24, 25). Similarly, a study by Zhang identified the rate of feeding advancement as an independent risk factor for achieving TEN in infants with 32 weeks ≤ GA < 34 weeks (odds ratio (OR) = 1.373, 95% confidence interval (CI): 1.262–1.493) (26). In addition, a declining trend in the average daily rate of feeding advancement was observed in Group A1 during weeks 3 and 4, and in Group B1 during weeks 2 and 3. These findings indicate that, in clinical practice at the study institution, the rate of feeding advancement may have been intentionally slowed as infants approached or achieved TEN. Although this approach may contribute to a longer time to reach TEN, its effect on reducing the incidence of NEC remains uncertain.

### 4.3 Effects of FI and BPD on time to achieve TEN

In this study, the incidence of FI was 52.55% (72/137), which was lower than the internationally reported rate of 75% (27). The incidence of FI in Group A1 was 75.76% (50/66), while that in Group A2 was 30.99% (22/71). The higher incidence of FI in preterm infants with GA < 32 weeks may be associated with immature gastrointestinal development and delayed meconium passage. Although the FI incidence rate was higher in Group A2 than in Group A1, the difference did not reach statistical significance. This may be attributed to variability in clinical decision-making among physicians, with differing levels of caution applied when managing enteral feeding in infants with GA < 32 weeks. In contrast, a statistically significant difference in FI incidence was observed between Groups B2 and B1, with a higher rate reported in Group B2. Both domestic and international studies have identified FI as an early clinical indicator of NEC and systemic infection (27, 28).

The presence of FI symptoms often prompts clinicians to reduce the rate of feeding advancement or to initiate temporary fasting due to concerns about progression to NEC, which can delay the achievement of TEN. At the study institution, feeding protocols in the NICU may have been overly conservative, a factor that warrants further evaluation.

In this study, cases of BPD were primarily concentrated in Group A, likely reflecting the greater severity of initial lung injury in preterm infants with lower GA. Milanesi et al. reported that infants diagnosed with BPD had lower mean GA and BW (29). The lower the gestational age, the higher the risk of BPD (30). Although early enteral and parenteral nutritional support plays a crucial role in lung development, injury and repair (31), clinicians tend to exercise greater caution in the feeding of preterm infants with a gestational age of < 34 weeks, especially those < 32 weeks, due to the high risk of unstable vital signs caused by underlying diseases, FI and other factors. This contradiction is also one of the reasons why the current feeding status of preterm infants in China lags behind international guidelines. In addition, the immature development of human milk banks and the low rate of human milk feeding in preterm infants may also increase the incidence of FI and hinder the smooth implementation of enteral feeding.

The results of this study showed that a higher incidence of BPD was observed in Group A2 compared to Group A1, indicating that the EN process may be more frequently disrupted in infants affected by BPD. Clinicians in domestic NICUs tend to be more cautious when feeding preterm infants with young gestational age, feeding intolerance, and multiple high-risk factors. The lack of human milk banks and the increasing prevalence of allergic diseases result in fewer preterm infants who can safely receive exclusive breastfeeding, which is the main cause of the delayed achievement of TEN in China. In this study, the initial feeding volume and feeding rate in the first two postnatal weeks of Group A2 were significantly lower than those of Group A1, with an ultimately higher incidence of BPD. Studies by Uberos et al., Bai XT, Ba RH (32–34) demonstrated that insufficient enteral nutrition energy intake in the first 2 weeks after birth is a risk factor for BPD, and prolonged time to achieve TEN is an independent risk factor for BPD in preterm infants with GA < 32 weeks.

Thiess et al., in a retrospective analysis, reported that insufficient energy intake from EN during the first 2 weeks of life in infants with GA < 32 weeks was associated with an increased risk of moderate to severe BPD (35). Milanesi et al. found that in the BPD group, enteral fluid and caloric intake were significantly reduced after the second postnatal week, with some infants exhibiting continued reduction through the fourth week (29). These findings align with the observed delay in achieving TEN in this study. Infants with BPD exhibit increased respiratory work and altered systemic blood distribution, which impair gastrointestinal perfusion, peristalsis, and digestive function. Meanwhile, their limited fluid tolerance leads to cautious feeding advancement, potentially delaying the achievement of TEN. In this study, fluid restriction without caloric limitation was implemented in infants with severe acute respiratory failure. However, limited by the study dataset, the causal relationship could not be fully confirmed, and the underlying mechanisms warrant further in-depth investigation. The incidence of RDS and rate of mechanical ventilation use in Group A2 were higher than those in Group A1, but the differences were not statistically significant. This may be attributed to the relatively small sample size, as well as the absence of data on RDS severity, PS administration frequency and dosage, and mechanical ventilation modes, which prevented a more accurate assessment of disease severity. The PS utilization rate in Group A2 was higher than that in Group A1, suggesting that the severity of primary pulmonary disease may be associated with the development of BPD.

Delayed attainment of TEN is associated with a corresponding prolongation of PN duration and an increased risk of cholestasis. Interrupted enterohepatic circulation, metabolic burden of nutritional solutions, and increased risk of recurrent infection (36–38) may impair hepatocyte function and lead to elevated direct bilirubin. The PN duration in Groups A2 and B2 was longer than that in Groups A1 and B1, and the overall incidence of cholestasis showed an upward trend with the prolonged PN duration, with no statistically significant difference observed. The reasons for the non-statistically significant difference in this study are as follows: ① The limited sample size (with a small total number of cholestasis cases) resulted in insufficient statistical power; ② In our center, a standardized strategy of “early initiation of enteral nutrition plus gradual PN tapering” is routinely implemented for infants receiving PN. Structured lipid emulsions are used as the lipid preparation; levocarnitine is administered when triglyceride levels increase significantly. For enteral nutrition, a formula with slightly higher caloric density is selected to minimize the duration and dosage of parenteral nutrition. Liver function is monitored regularly, and the nutritional formula is adjusted accordingly, which has reduced the incidence of severe cholestasis to a certain extent.

## 5 Conclusion

Preterm infants with GA less than 32 weeks are at increased risk of developing BPD due to immature lung development and initial pulmonary injury. Inadequate early caloric intake from enteral feeding may impair lung growth, hinder injury recovery, and limit alveolar development, further exacerbating BPD risk. Conversely, infants already diagnosed with BPD may exhibit reduced fluid tolerance, leading to feeding volume restrictions and delayed achievement of TEN. Currently, a nutritional strategy of fluid restriction without caloric limitation is adopted to address this issue. This study has several limitations, including its single-center design, relatively small sample size, absence of body length and head circumference data, and lack of detailed analysis regarding the effects of different milk products. In addition, the sample sizes of subgroups including extremely preterm infants with gestational age ≤28 weeks and small for gestational age (SGA) infants were relatively small, and stratified analysis and sensitivity analysis were not performed. Furthermore, nutritional strategies are affected by infants’ respiratory and circulatory status. Due to space limitations, this study only briefly presented respiratory-related variables (respiratory support, PS administration) and circulatory-related variables (hsPDA), but multivariate confounding adjustment for more detailed quantitative indicators was not conducted, which may lead to confounding bias. Despite these limitations, the findings indicate that the rate of enteral feeding advancement in preterm infants with GA less than 34 weeks is slower than the rates recommended by current clinical guidelines. Such issues may be present in other Grade A tertiary hospitals. Early initiation of feeding, higher initial feeding volumes, and a more proactive feeding approach during the first two postnatal weeks may contribute to earlier achievement of TEN and reduction in related complications.

## Data Availability

The original contributions presented in the study are included in the article/supplementary material, further inquiries can be directed to the corresponding author.
